# Alloparenting for chimpanzee twins

**DOI:** 10.1038/srep06306

**Published:** 2014-09-09

**Authors:** Takeshi Kishimoto, Juko Ando, Seiki Tatara, Nobuhiro Yamada, Katsuya Konishi, Natsuko Kimura, Akira Fukumori, Masaki Tomonaga

**Affiliations:** 1 Faculty of Liberal Arts, University of the Sacred Heart, Tokyo, Japan; 2 Faculty of Letters, Keio University, Tokyo, Japan; 3 Noichi Zoological Park of Kochi Prefecture, Kochi, Japan; 4 Hirakawa Zoological Park, Kagoshima, Japan; 5 Primate Research Institute, Kyoto University, Aichi, Japan

## Abstract

In April 2009, a female chimpanzee named Sango, living in a captive group at the Noichi Zoo, Japan, gave birth to dizygotic male-female twin chimpanzees (male: Daiya, female: Sakura). The extent to which adult group members cared for the twins was investigated using a focal animal sampling method targeting six adults (one male) when the twin chimpanzees were two years old. Data were collected for an average of 6.78 h (*SD* = 0.79) per focal participant. An unaffiliated female adult of Sango was engaged in parenting Sakura as much as Sango. Given that Sakura was in lesser proximity to Sango than Daiya, Sakura's departures from her mother and her ability to gesture requests might have enabled non-kin adults to provide her care.

In humans (*Homo sapiens*), having young twins sometimes has a deleterious effect on the emotional well-being of mothers. Thorpe, Golding, MacGillivray, and Greenwood^1^ showed that mothers of twins at 5 years had significantly higher malaise scores, indicative of depression, than did mothers of singletons at the same age. Maternal depression is at least in part caused by the heavier burden of parenting on mothers of twins than on mothers of singletons^2^,^3^. This occasionally results in maltreatment of twins^4^. Diminishing the parental stress of the mothers of twins might require the involvement of alloparents who share the burden of parenting with mothers such as fathers, extended families, and community members^5^.

Chimpanzees (*Pan* *troglodytes*), the closest relatives of humans, normally give birth to one infant at a time; however, they also give birth to twins, albeit infrequently. According to the data from the International Species Information System (ISIS), one of 59 captive births was multiple^6^. Some cases of twin births have also been reported in the wild. For example, Matsumoto-Oda^7^ reported a case of twin birth among the chimpanzees of the Mahale Mountain National Park, Tanzania. Additionally, Goodall^8^ reported a case of twin birth in the Gombe National Park, Tanzania. However, in both these cases, the twins were unable to reach adulthood, as one or both the twins died within few years^7^,^8^. Even among captives, 64% of individuals born as twins die by the end of the first year of their birth, whereas only 23% of singletons pass away during the first year of life^6^.

One of the difficulties in rearing infant twins among chimpanzee mothers might have been the absence of alloparents. This idea is supported by the single case of successfully raised chimpanzee twins in the wild at Gombe. The mother, Gremlin, gave birth to female twins (Golden and Glitter) in 1998^9^. The twins are still alive and surviving well into adulthood^10^. The Jane Goodall Institute^10^ has introduced video episodes indicating that the twins were often taken care of by their older sister Gaia. These episodes suggested that alloparenting by Gaia reduced the parental burden on Gremlin, and thus allowed for the successful rearing of the twins. Although reports from the Jane Goodall Institute supported the idea that chimpanzee mothers can rear twins if alloparents are available, quantitative data remain sparse. In particular, no research has indicated who is more suitable to become a chimpanzee alloparent or the types of care responsibilities assumed by alloparents.

In April 2009, a female chimpanzee named Sango living in the captive group at the Noichi Zoological Park of Kochi Prefecture, Japan, gave birth to dizygotic male-female twins (male: Daiya, female: Sakura; Figure 1). To date, the twins have been successfully reared by their mother. To investigate the hypothesis that alloparents are necessary for twin rearing, we observed the chimpanzee group at Noichi when the twins were two years old. In general, the third year of life in chimpanzees is the middle of infancy in their life cycle, and they still depend on their mothers in diverse ways. For example, infant chimpanzees still depend on their mothers for transport by riding on their mothers when they travel^8^,^11^. In other words, to ensure their infants' survival, chimpanzee mothers must take care of them. We investigated whether and how adult group members (besides the mother) cared for the twins. Additionally, we explored the causes of alloparenting among chimpanzees, focusing on two primary factors, namely, the individuals that were most likely to become alloparents and the external triggers for alloparenting behavior.

## Results

### Parenting behavior among adult chimpanzees

**Figure 2:**
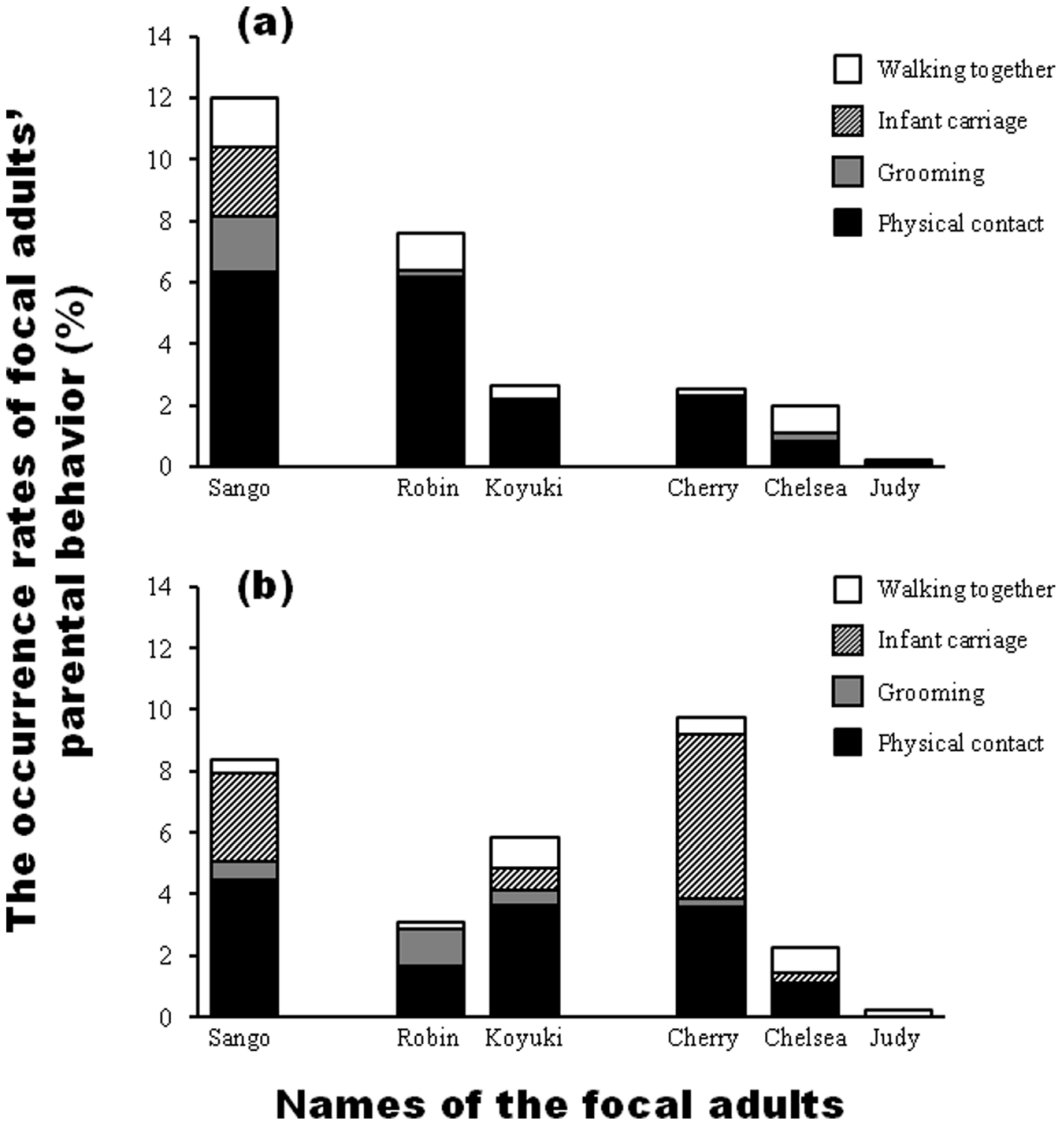
The occurrence rates of parental behaviors by each focal adult toward Daiya (a) and Sakura (b). Sango is the mother of Daiya and Sakura. Robin and Koyuki were adults affiliated with Sango. Cherry, Chelsea, and Judy were adults unaffiliated with Sango.

Figure 2(a) shows the occurrence rates of parenting behavior of focal adults toward Daiya, the male twin. Sango had the highest parenting behavior rate of all adult chimpanzees and was engaged in all four kinds of parenting behaviors (walking together, infant carriage, grooming, and physical contact). Sango was the only adult who was observed carrying Daiya. Robin, the father of Daiya and one of Sango's affiliated adults, was also engaged in parenting, particularly in physical contact with Daiya. The other four female adults cared for Daiya at a lower rate than did Sango and Robin.

Figure 2(b) shows the occurrence rate of parenting behavior of focal adults toward Sakura, the female twin. Unlike in the case of Daiya, Cherry, who was one of Sango's unaffiliated adults, was engaged in parenting Sakura for as much time as did Sango. Additionally, Sango, Cherry, and Koyuki (an adult affiliated to Sango) were engaged in all four kinds of parenting behavior. Moreover, besides Sango, three adult females (Koyuki, Cherry, and Chelsea) were found carrying Sakura.

### The effect of proximity between focal adults and Sango, and focal adults and twins on parenting behavior

To determine the effects of the social relationships of the twins with Sango on the parental behavior by focal adults other than Sango, we conducted correlation analyses on proximity rate between twins and focal adults, Sango and focal adults, and the occurrence rate of alloparental behavior by focal adults.

There was no significant correlation between the occurrence rate of parental behavior toward Daiya by focal adults and the proximity rate between Sango and the focal adults. However, there was a positive correlation between the occurrence rates of parental behavior toward Daiya by focal adults and proximity between Daiya and the focal adults (Table 1). The same was true for Sakura: There was no significant correlation between the occurrence rate of parental behavior toward Sakura by focal adults and the proximity rate between Sango and the focal adults. However, there was a positive correlation between the occurrence rates of parental behavior toward Sakura by focal adults and proximity between Sakura and the focal adults (Table 2).

### The effect of proximity between Sango and the twins on proximity between focal adults and twins

Table 3 shows the cross-tabulation of the number of scans revealing the relations between whether Daiya and his mother Sango were within the reach of each other and whether each focal adult and Daiya were simultaneously within the reach of each other. In this analysis, we counted the total scans conducted when the adult chimpanzee was in the outside enclosure, and categorized the scans into four conditions that occurred in a combination of whether Daiya and his mother, Sango, were within each other's reach, and whether each focal adult and Daiya were simultaneously within each other's reach. For example, the total number of scans conducted when Robin was in the outside enclosure was 382, and for 32 of these scans, both Sango and Robin were within reach of Daiya, for 114 scans Sango was within reach but Robin was out of reach of Daiya, for 62 scans Sango was out of reach but Robin was within reach of Daiya, and for 174 scans both Sango and Robin were out of reach of Daiya.

Koyuki and Daiya were within the reach of each other at a higher rate when Sango and Daiya were also within the reach of each other than when they were out of reach (χ^2^(1) = 6.12, *p* = 0.013). In contrast, Chelsea and Daiya were within the reach of each other at a higher rate when Daiya and Sango were not within the reach of each other (χ^2^(1) = 16.39, *p* < 0.00006).

Table 4 shows the cross-tabulation of the number of scans revealing the relations between whether Sakura and her mother were within the reach of each other and whether each focal adult and Sakura were simultaneously within the reach of each other. All of Sango's affiliated adults (Robin and Koyuki) and Sakura were within the reach of each other at a higher rate when Sango and Sakura were also within the reach of each other (Robin: χ^2^(1) = 8.52, *p* < 0.004; Koyuki: χ^2^(1) = 4.31, *p* < 0.038). In contrast, two of Sango's non-affiliated adults (Cherry and Chelsea) and Sakura were within the reach of each other at a higher rate when Sakura and Sango were not within the reach of each other (Cherry: χ^2^(1) = 22.36, *p* < 0.000002; Chelsea: χ^2^(1) = 11.95, *p* < 0.001).

### Communication between focal adults and twins before infant carriage

By using the 1-min fixed-interval point sampling method, we recorded infant carriage at 50 sampling points. We observed communications between focal adults and infants before four of these 50 sampling points. All four communications were between Sakura and focal adults. Two were adult-initiated communications between Koyuki and Sakura, and Sango and Sakura. The other two were infant-initiated communications between Cherry and Sakura, and Chelsea and Sakura. Both Cherry and Chelsea were Sango's non-affiliated adults. The details are as follows.

#### Adult-initiated communication between Koyuki and Sakura

**Figure 3:**
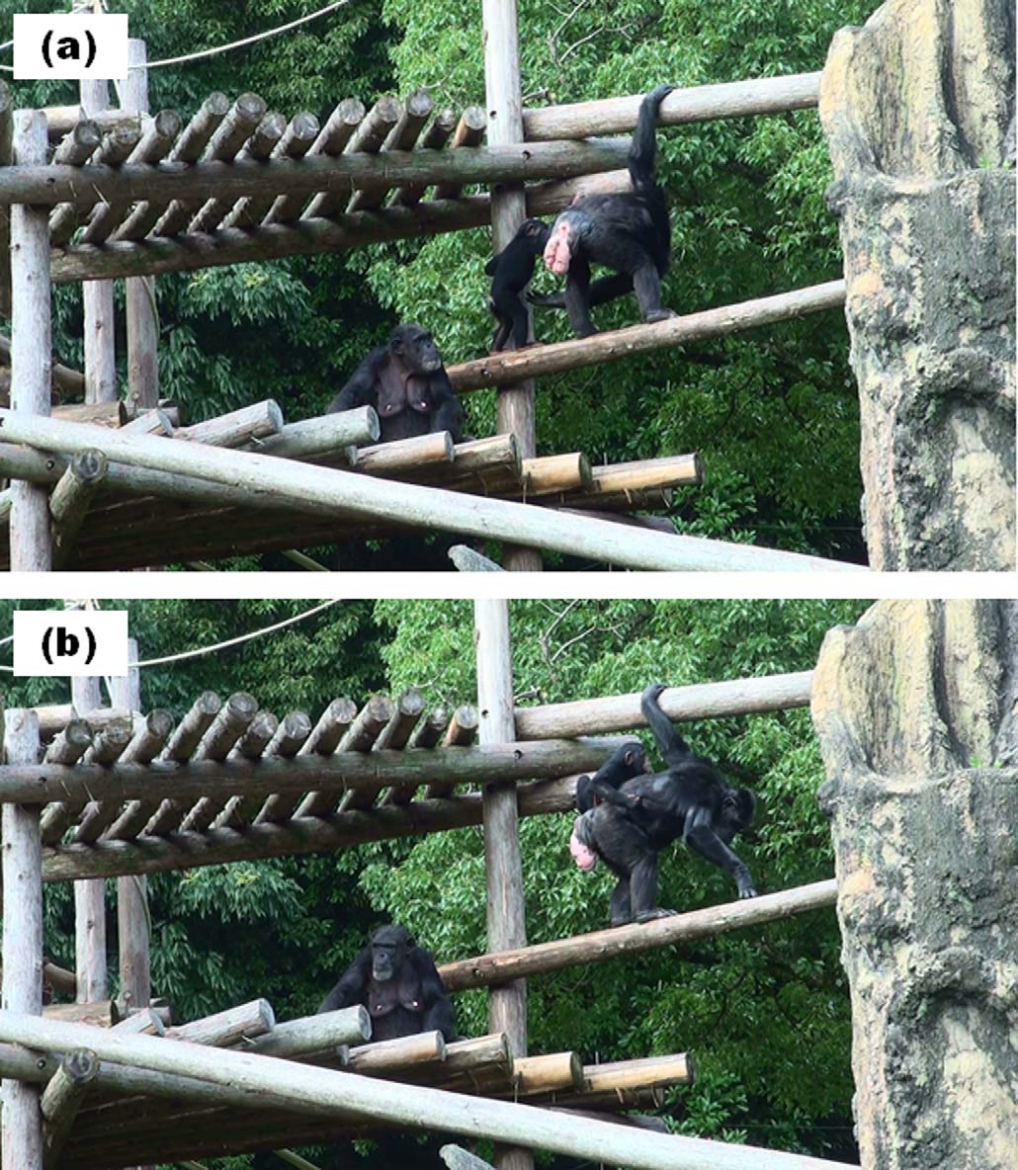
Adult-initiated communication. Koyuki stretched her right hand toward Sakura (a). Then Sakura climbed on to Koyuki's back (b). Photo courtesy Takeshi Kishimoto.

At 15:32 h on July 23, 2011, Sakura and Koyuki were in the proximity to Sango. When Koyuki started to move away, Sakura gently touched Koyuki. Thereafter, Koyuki extended her right hand to Sakura with her palm facing up (Figure 3a). Next, Sakura approached Koyuki and climbed onto Koyuki's back (Figure 3b).

#### Adult-initiated communication between Sango and Sakura

**Figure 4:**
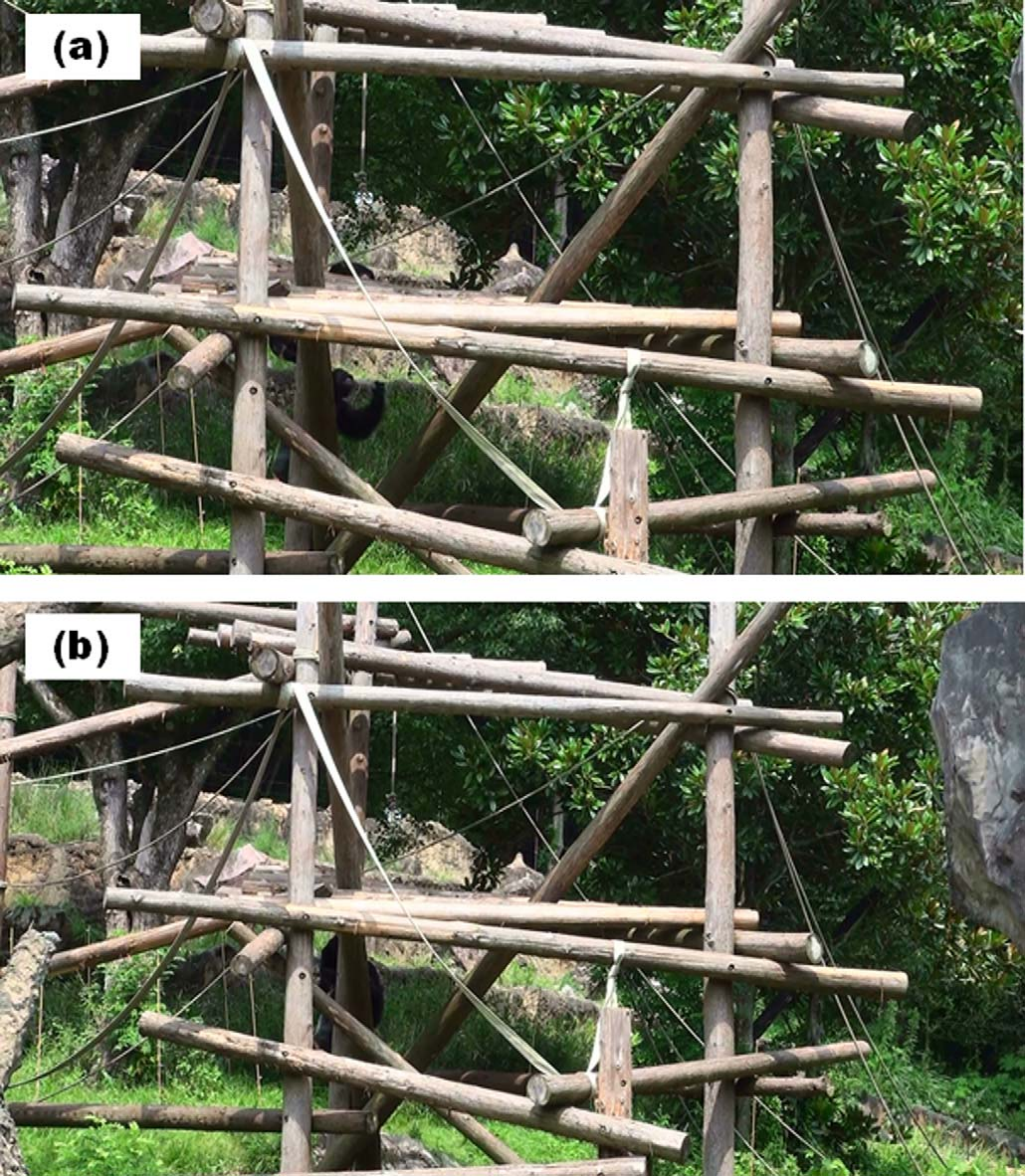
Adult-initiated communication. At the lower part of the tower, Sango stretched out her left hand (a). Then Sakura fell into Sango's arms (b). Photo courtesy Takeshi Kishimoto.

At 13:22 h on August 18, 2011, Sango began to move under the tower, and Daiya and Sakura followed. When Sango reached the lower step, Sango directed her gaze to Sakura and stretched her hand toward Sakura (Figure 4a). Thereafter, Sakura fell into Sango's arms and climbed onto Sango's back (Figure 4b). Daiya went to Sango's and Sakura's side.

#### Infant-initiated communication between Cherry and Sakura

**Figure 5:**
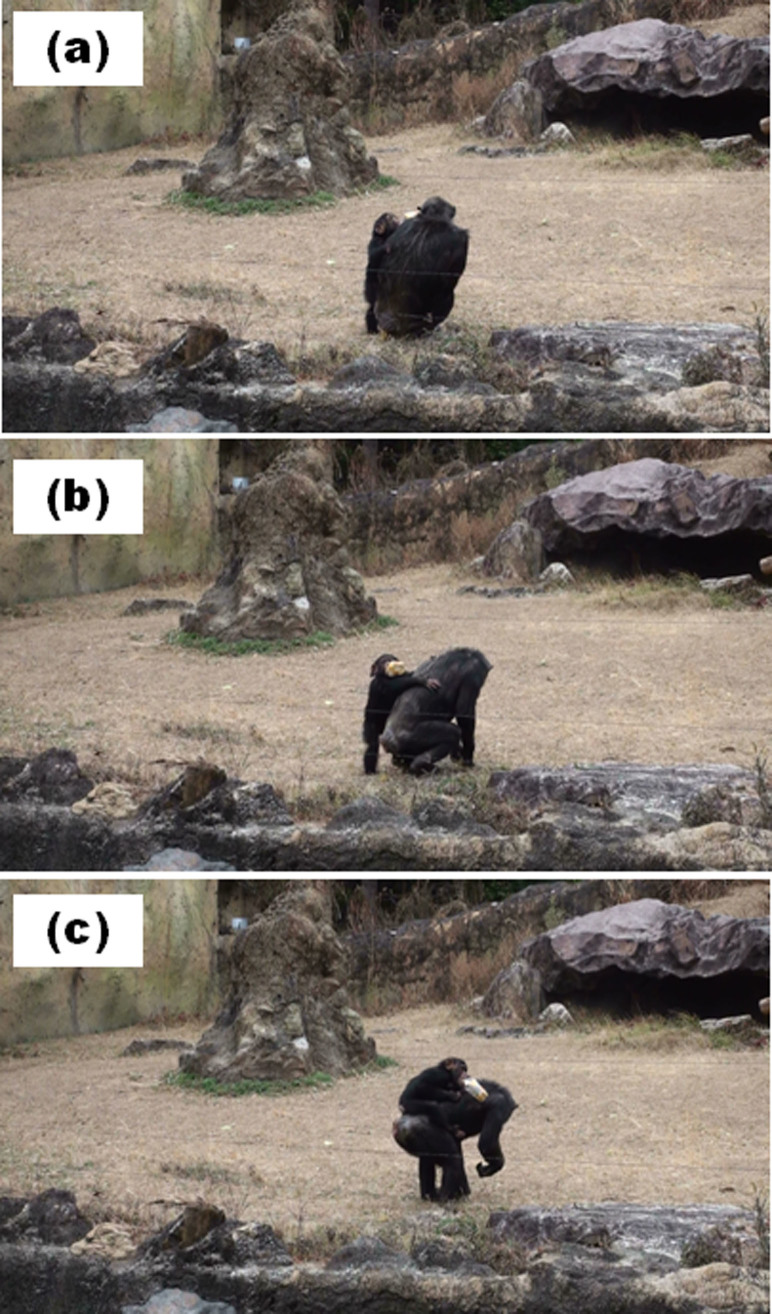
Infant-initiated communication. Sakura roughly touched Cherry's head (a). Then Cherry shifted her posture (b), and Sakura climbed upon Cherry's back (c). Photo courtesy Takeshi Kishimoto.

At 11:58 h on February 5, 2012, Cherry and Sakura were resting about 2 m apart. After Sakura approached the wall and kicked it, she approached Cherry and roughly touched Cherry's face with her left hand (Figure 5a). Thereafter, Cherry shifted her posture and moved (Figure 5b), and Sakura climbed on Cherry's back (Figure 5c).

#### Infant-initiated communication between Chelsea and Sakura

**Figure 6:**
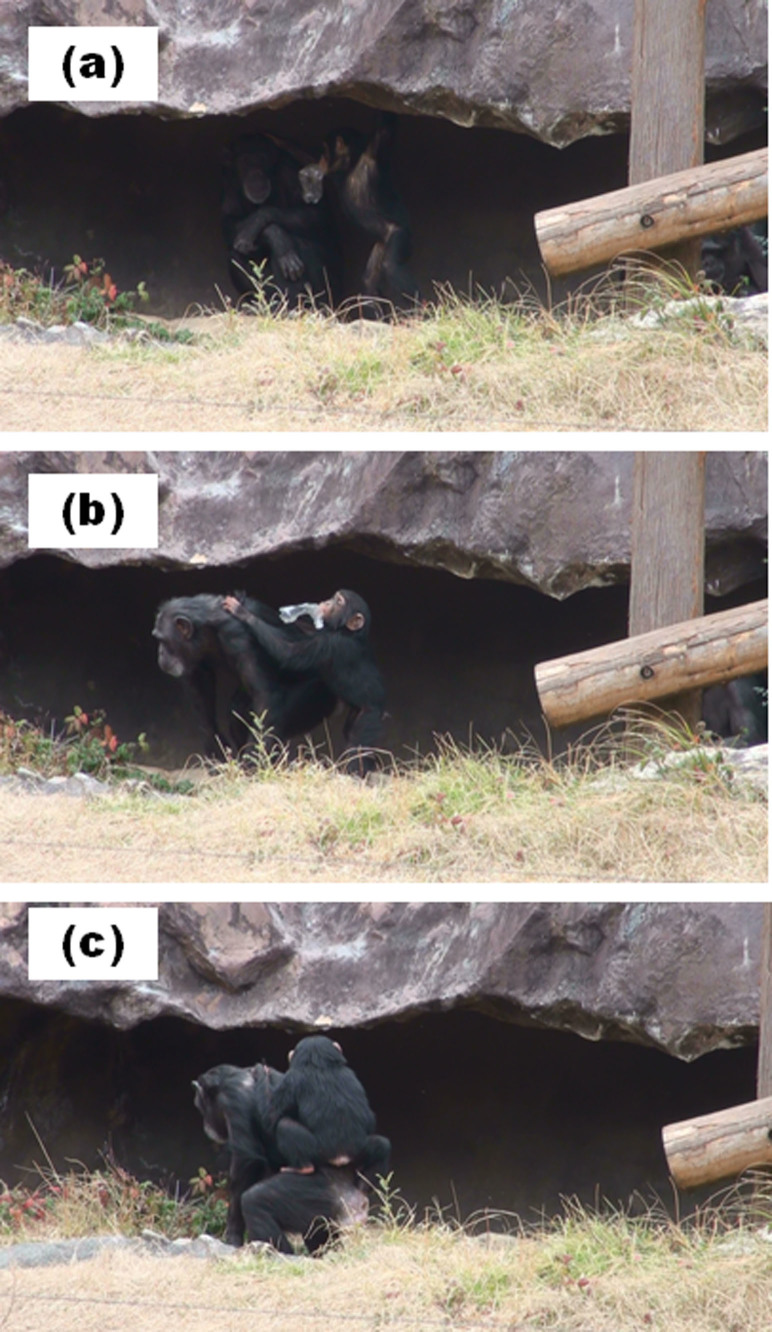
Infant-initiated communication. In the cave, Sakura touched Chelsea's head (a) and dragged Chelsea outside (b). Then Sakura climbed upon Chelsea's back (c). Photo courtesy Takeshi Kishimoto.

At 0: 30 h on February 5, 2012, Chelsea, Sakura, and Cherry were resting in the cave with about 1 m of distance among them. Sakura approached Chelsea, touched Chelsea's head, and dragged Chelsea outside the cave (Figure 6a, b). Thereafter, Chelsea moved, and Sakura climbed on Chelsea's back (Figure 6c).

### Proximity between Sango and the twins

**Figure 7:**
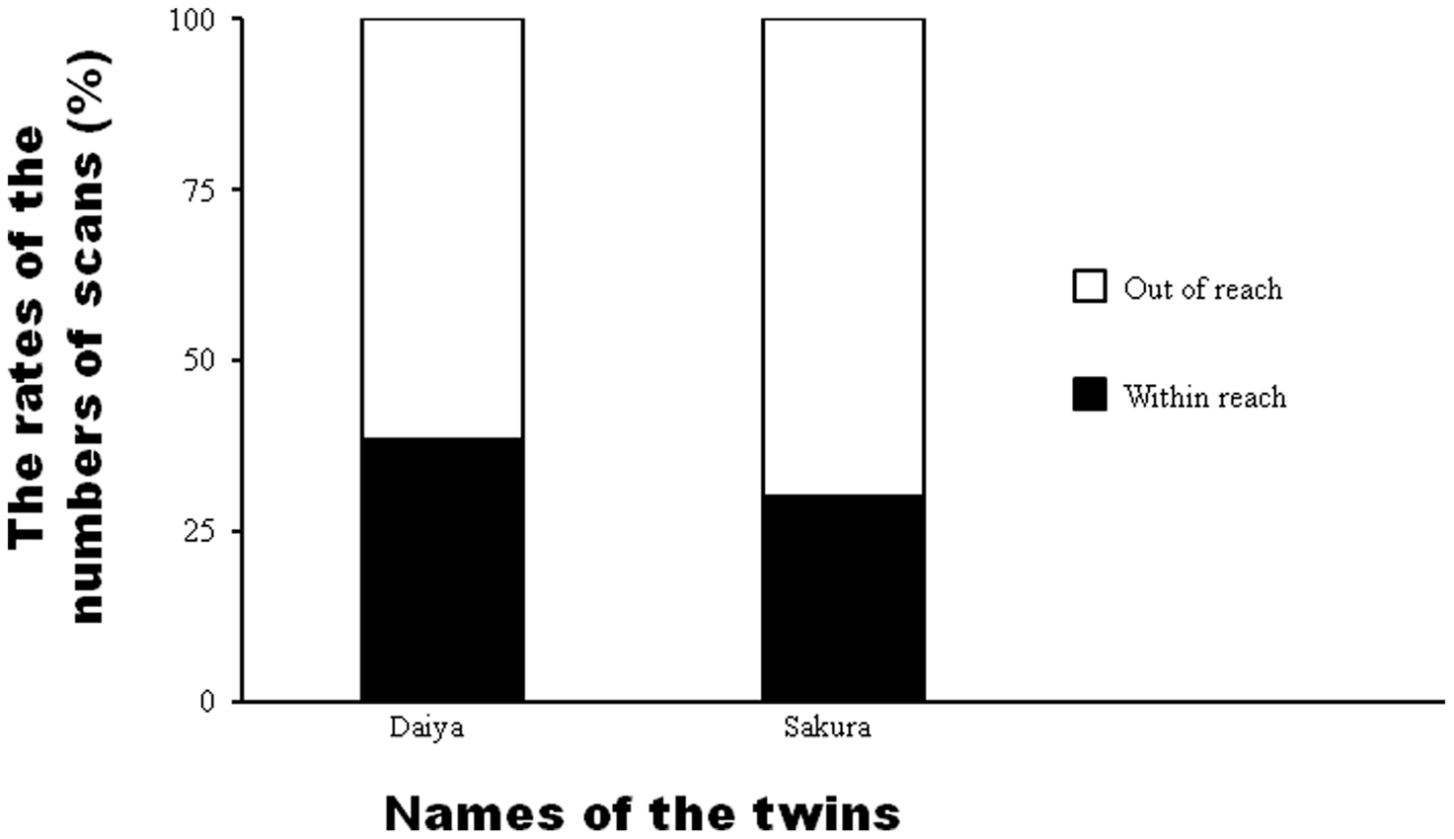
The rates of proximity and non-proximity scans between Sango and each twin.

To determine whether Sango was equally close to each of her twins, we compared the numbers of proximity and non-proximity scans with Sango and each of Daiya and Sakura by using a chi-squared test. Daiya was close to Sango at a significantly higher rate than was Sakura (Figure 7; χ^2^(1) = 5.59, *n* = 764, *p* < 0.018).

## Discussion

Twin rearing is rare in chimpanzees, and thus, very few studies have investigated how twin chimpanzees are reared in their communities. To our knowledge, this is the first study to provide quantitative data on parenting behaviors by a mother chimpanzee and adult group members toward infant twins.

Rearing twins would be difficult for chimpanzees because of the heavier parenting burden on mothers. In fact, reports of successful twin rearing by chimpanzees are scarce. We hypothesized that alloparents, who share the burden of parenting, would be necessary for successful rearing of twins.

In the captive community of chimpanzees at the Noichi Zoological Park, we found that parenting behaviors were observed in not only the mother of the twins, but also the adult group members. In the case of Daiya—the male twin—mother Sango and father Robin were particularly engaged in parenting. The aspects of their parenting behaviors, however, were different. Sango was engaged in all four kinds of parenting behavior, while Robin was primarily engaged in physical contact and walking together. This suggests that parenting responsibilities were divided between Robin and Sango. While Sango provided essential care for Daiya's survival (e.g., infant carriage), Robin and Daiya were often engaged in play. The physical contact and walking together by Robin toward Daiya might have been facilitated by the types of social play common in chimpanzees such as “play bite” and “play run”^14^. However, the observation of play between the infant male chimpanzee and the adult male was unremarkable. According to Lansdorf et al.^15^, infant male chimpanzees in the Gombe National Park were engaged in social interaction with adult males to a greater extent than infant female chimpanzees. This suggests that the preference for adult males is a general trait among infant male chimpanzees. Thus, the parenting of Daiya by Sango and Robin would be considered normal chimpanzee behavior.

In the case of Sakura, the female twin, not only Sango, but also Cherry and Koyuki were engaged in parenting. Interestingly, these three adult female chimpanzees provided all four kinds of parenting behavior. This indicates that adult females (Cherry and Koyuki) provided the same care as the mother, suggesting that they were alloparents of Sakura. Perhaps such alloparenting by Cherry and Koyuki reduced the burden of parenting for Sango, thus facilitating successful twin rearing by Sango.

Previous studies have reported cases of alloparenting in chimpanzees. Most were the cases of adoption, in which alloparenting was conducted by adults other than mothers after the mother had died. Boesch, Bole, Eckhardt, and Boesch^16^ reported that not only kin female adults but also non-kin adults adopted orphaned infants and provided essential care for the infants' survival, such as food sharing or infant carriage. On the other hand, Wroblewski^17^ reported a case of adoption of an infant by his grandmother when the mother was alive. This was the first report of chimpanzee adoption from a living mother. These examples suggest that most alloparenting behaviors toward infants were conducted after the infants' mothers had died. The parenting behaviors of other adults are rare when mothers are still alive. Furthermore, such examples have been limited to care by kindred adults. Unlike the findings of these previous studies, our study represents the first example where an infant was cared for by non-kin adults while the infants' mother was still alive.

Why did female adults, such as Cherry and Koyuki, care for Sakura despite there being no kinship between them? One possibility is that alloparenting toward infants by non-kin adults is altruistic behavior toward the mothers of the infants. Stanford^18^ reported that female capped langurs (*Prebytis pileatus*) often provide care for non-kin infants. He suggested that these allomothering behaviors by females might have adaptive altruistic behavior among group females, in that it enables lactating females to increase the feeding time. Our results, however, did not support this idea because of the following points. If the allomothering behaviors in this study represented altruistic behavior from Cherry or Koyuki toward Sango, a long-lasting relationship must have existed between them such that the altruistic behaviors would be rewarded in the form of returned altruistic behavior from the recipient^19^. However, we found no significant relation between each adult's parenting behavior occurrence rates toward Sakura and the index of intimacy between Sango and each adult as measured by the proximity rate between Sango and each adult. Additionally, the focal adult who provided the most care for Sakura was Cherry, who was one of Sango's non-affiliated adults. These results suggest that the alloparenting behavior toward Sakura by other adult group members was not due to the bonding between Sango and the adults, suggesting that the altruistic rewards from Sango toward the adults were unlikely to occur.

Another hypothesis is that Sakura herself built social relationships with Cherry and Koyuki, and thus procured parenting behaviors from them. This idea was supported by our results. First, the occurrence rate of parenting behaviors toward Sakura from focal adults besides Sango were positively correlated with the index of intimacy between Sakura and each adult, which was measured by the proximity rate between Sakura and each adult. This suggests that Sakura was provided care by adults who were familiar with her. Second, Sakura was close to Cherry—one of her alloparents—at a higher rate when Sango was *not* close to them. This suggests that when Sakura was free from Sango, she would move toward Cherry. Thus, Sakura independently built her own altruistic relationships with group adults, including Cherry, who were unfamiliar with Sango. In some primate species, whether infants build their own altruistic relationships or remain close to their mothers depends on their mothers' rearing style (so-called “maternal style”). In Japanese macaques (*Macaca fuscata*), infants are often handled by adults other than mothers when the mothers of the infants are not protective^20^,^21^. Such infants do not make physical contact with their mothers or are not groomed by their mothers; thus, these infants are handled by adults attracted by them^20^. This relation between maternal style and the frequency of infants being handled by adults other than the mothers was observed in not only macaques but also chimpanzees. According to Hemelrijk and de Kogel^22^, chimpanzee infants whose mothers often broke contact spent more time in social play with others and less time in contact with their mothers. In our study, Sakura (30.10%) was close to her mother Sango at a slightly lower rate than was Daiya (38.22%). This would be because those infants were twins and could not be cared for equally by Sango, and Sango cared for Daiya on a priority basis. Consequently, Sakura would be slightly less close to her mother, leading her to come free, as was observed by Hemelrijk and de Kogel^22^. Sango's inability to provide equal care might have made Sakura more sociable.

Some questions remain unresolved by the present study. First, we could not entirely reveal why adults other than Sango provided care for Sakura. Previous experimental studies have indicated that chimpanzees do not take care of others' welfare^23^. Thus, it is puzzling that other adults would have engaged in taxing parenting behaviors (e.g., infant carriage). This question could be answered, at least in part, by the communication between Sakura and alloparents. We observed not only adult-initiated communication, which was also observed by Hirata^24^, but also infant-initiated communication before infant carriage. We observed that Sakura dragged Cherry or Chelsea before she climbed upon their backs, as if she had requested carriage from them (Figures 5 and 6). Yamamoto, Humle, and Tanaka^25^ revealed that chimpanzees help others when the recipients express their requests by reaching toward what they want. This suggests that chimpanzees help others when others express their needs by gestures. Sakura's pulling of Cherry or Chelsea would facilitate their understanding of Sakura's requests, and accordingly, they provided care for her. Interestingly, infant-initiated communication, such as dragging adults, was not observed between Sakura and her mother. Moreover, Daiya was never engaged in such communicative behavior toward Sango. Such infant-initiated gestures are possibly only necessary when infants request parenting from adults other than their mothers. Human infants who are unable to express their requests using speech typically use pointing gestures to not only mothers but also other adults (e.g., nursery staff^26^). Taken together, the gestural expressions of requests by infants would be important for both species in procuring alloparental care from adults.

Another question that remained unaddressed was the benefit of becoming an alloparent. What benefit would alloparents such as Cherry and Koyuki derive from their altruistic care for Sakura? Perhaps those alloparents would derive benefit as Sakura grows older. Thus, longitudinal observations are necessary to reveal any potential benefits for these alloparents.

In conclusion, this is the first study suggesting that alloparenting from adults other than mothers would contribute to the successful rearing of twins in captive chimpanzees. We also suggested that infants' departing from mothers and gesturing their own requests would make non-kin adults provide care for the infants. Our results provide some insight into why humans give birth in short calving intervals, and how institutions of human alloparenting (e.g., nursery school) originated.

## Methods

### Participants

The present study was conducted between April 2011 and March 2012 on a captive social group of chimpanzees at the Noichi Zoological Park of Kochi Prefecture, Japan. In April 2011, the study group consisted of 10 chimpanzees, including two adult males, six adult females, and the dizygotic male–female twin infants (Table 5). Of these, one adult male (Gou) and one adult female (Maya) were often kept in indoor rooms, and there were few opportunities to observe them in the outdoor enclosure. Thus, we excluded them from the participants.

Daiya, the male infant, and Sakura, the female infant, were the two-year-old twins. They were born on April 18, 2009, to Sango, a 35-year-old wild-born female. The father of the twins was Robin, a 15-year-old captive-born male. The birth of the twins was natural, and there are not any human interventions.

According to the breeding record published on the website of Noichi Zoological Park of Kochi Prefecture^27^, Sango and twins appeared in the outdoor enclosure for the first time on April 27, 2009. After that, other adult group members sometimes touched the twins in Sango's arms. From around December 2009, after the twins were 8 months old, Koyuki, Robin, and Cherry were sometimes observed holding the twins in their arms. According to Ichino^28^ who investigated the social relationship of this captive social group of chimpanzees, when the twins were aged between 18 and 23 months, Daiya spent significant amounts of time with Sango, Sakura, and Robin, while Sakura spent much time with Sango, Daiya, and Cherry.

The research protocol was approved by a committee of Noichi Zoological Park of Kochi Prefecture and by the AnimalWelfare and Animal Care Committee of KUPRI and by the Animal Research Committee of Kyoto University. All procedures adhered to the Ethical Guidelines for the Conduct of Research on Animals by Zoos and Aquariums issued by the World Association of Zoos and Aquariums (WAZA), the Code of Ethics issued by the Japanese Association of Zoos and Aquariums (JAZA), and the Japanese Act on Welfare and Management of Animals. This study was carried out in accordance with these guidelines.

### Data collection

In this study, the first author (TK) conducted observation of six adult chimpanzees by using a 10-min focal animal sampling procedure^12^ when the group members were in the outside enclosure (853 m^2^). Observations were made between 10:00 and 17:00 h on the days when Sango and twins were in the outside enclosure. A video camera (Sony HDR-CX560V) was used to record the behavior of focal participants. The focal observations were conducted in a predetermined order. Observations were not conducted during the feeding time or when it rained.

Parenting behaviors were recorded using 1-min fixed-interval point sampling^12^. Parenting behaviors were categorized into the following four mutually exclusive behaviors: *walking together*, which included focal adults walking with the twins while maintaining a distance of less than 1 m; *infant carriage*, which included cases in which the focal adults held the twin infants on their back or in ventral position while moving around; *grooming*, which included cases in which either focal adults groomed the twins or twins groomed the focal adults; and *physical contact*, which included cases of breast-feeding, or cases in which the body parts of the focal adults made contact with the body parts of infants, but excluded carriage and grooming instances. If infant carriage was noted, we recorded whether communication between the focal adults and infant twins occurred during the minute preceding the sampling point in which infant carriage was recorded. Adult–infant communication was classified into adult-initiated communication, which included cases in which focal adults outstretched their arm toward the infant; and infant-initiated communication, which included cases in which infants dragged the focal adults. Because there was a possibility that vocalizations of chimpanzees did not occur for communication but were a result of emotional arousal^13^, we did not define the screams or whimper calls by infants as infant-initiated communication. Since these behaviors were distinct, reliability tests were not conducted. Data were collected for an average of 6.78 h (*SD* = 0.79) per focal participant.

Besides focal observations, TK conducted scan sampling observations of all chimpanzees, including the six adults and the twins^12^. TK recorded participants who were within the reach of one another by taking notes. The two scan samplings were conducted at intervals of at least 10 min. TK did not conduct scan sampling while he was conducting the focal observation. A total of 382 scan samples were collected.

Table 6 shows the details about the information on the number of focal observation, observation days, observation time (min), and number of scans.

### Analysis

Proximity values (%) between chimpanzees A and B were calculated as follows: the number of scans in which A and B were within the reach of each other was divided by the total number of scans obtained on the observation days in which both A and B were in the outdoor enclosure and then multiplied by 100. The average proximity value between Sango and other adults was 11.91%. We defined Sango's affiliated and unaffiliated adults on the basis of these proximity scores. Adults with above-average proximity values were considered affiliated, whereas those with below-average proximity values were considered unaffiliated (Table 5).

The occurrence rates (%) of parenting behavior by adults were calculated as follows: the number of sample points in which the focal adults were observed engaging in one of the four kinds of parenting behavior was divided by the number of total sample points for the focal adults and multiplied by 100.

## Author Contributions

T.K., J.A. and M.T. contrived the observation. T.K. conducted the observation and analysed the data. T.K., J.A., S.T., N.Y., K.K., N.K., A.F. and M.T. discussed the results, and T.K. wrote the paper.

## Figures and Tables

**Figure 1:**
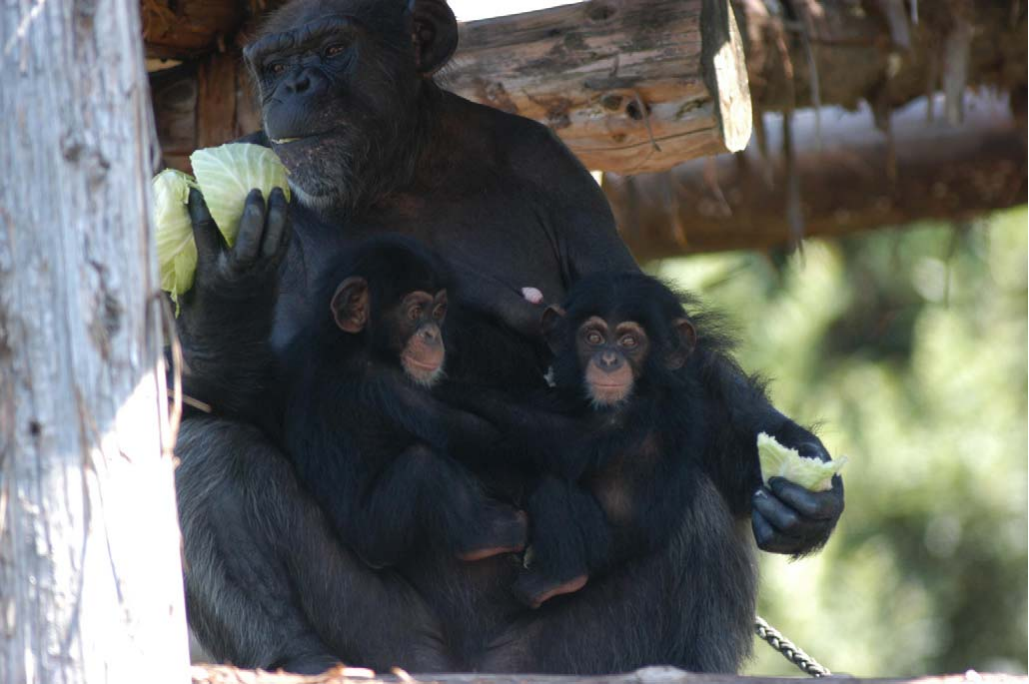
Sango was holding the twins (left: Daiya, right: Sakura) in her arms when they were 5 months old. Photo courtesy of Nobuhiro Yamada.

**Table 1:** Proximity and Parenting Behavior Occurrece Correlations for Daiya (n = 5)

	1.	2.	3.
1. Alloparenting toward Daiya	-	0.90*	0.80
2. Proximity rate with Daiya		-	0.60
3. Proximity rate with Sango			-

*Note.* Values in the table are Spearman's rank-order correlation coefficients.

*: *p* < .05.

**Table 2:** Proximity and Parenting Behavior Occurrece Correlations for Sakura (n = 5)

	1.	2.	3.
1. Alloparenting toward Sakura	-	0.90*	0.60
2. Proximity rate with Sakura		-	0.30
3. Proximity rate with Sango			-

*Note.* Values in the table are Spearman's rank-order correlation coefficients.

*: *p* < .05.

**Table 3:** Simultaneous Proximity between Sango and Daiya, and Daiya and Focal Adults

	Sango and Daiya		
	Within reach	Out of reach	χ^2^(1)
Robin and Daiya			
Within reach	32	62	0.92
Out of reach	114	174	
Koyuki and Daiya			
Within reach	32	30	6.12*
Out of reach	101	189	
Cherry and Daiya			
Within reach	9	26	2.84
Out of reach	120	177	
Chelsea and Daiya			
Within reach	7	45	16.39**
Out of reach	107	139	
Judy and Daiya			
Within reach	13	19	0.16
Out of reach	117	199	

*p < .05.

**p < .01.

**Table 4:** Simultaneous Proximity between Sango and Sakura, and Sakura and Focal Adults

	Sango and Sakura		
	Within reach	Out of reach	χ^2^(1)
Robin and Sakura			
Within reach	25	28	8.52**
Out of reach	90	239	
Koyuki and Sakura			
Within reach	29	44	4.31*
Out of reach	76	203	
Cherry and Sakura			
Within reach	13	101	22.36**
Out of reach	78	140	
Chelsea and Sakura			
Within reach	5	45	11.95**
Out of reach	86	162	
Judy and Sakura			
Within reach	12	30	0.14
Out of reach	96	210	

*p < .05.

**p < .01.

**Table 5:** Profiles of chimpanzees at the Noichi Zoological Park of Kochi prefecture

Name	Sex	Date of birth	Age^a^	Proximity value (%) with Sango^b^	Relationship with Sango^c^	Notes
Sango	F	1976^d^	35	-	-	Wild-born. The mother of twins (Daiya and Sakura)
Robin	M	Nov.12, 1995	15	16.75	Affiliated	Captive-born. Introduced to the Noichi group in 2008 with Sango and Koyuki. The father of the twins.
Koyuki	F	Apr. 30, 1993	18	26.70	Affiliated	Captive-born and introduced to the Noichi group in 2008 with Sango and Robin.
Cherry	F	1978^d^	33	6.63	Unaffiliated	Wild-born and introduced to the Noichi group in 1999.
Chelsea	F	Dec. 20, 1987	23	4.03	Unaffiliated	Captive-born and introduced to the Noichi group in 1994.
Judy	F	Jan. 5, 1990	21	5.46	Unaffiliated	Captive-born. An original member of the Noichi group.
Daiya	M	Apr. 18, 2009	2	-	-	One of the dizygotic twin children of Sango; father is Robin.
Sakura	F	Apr. 18, 2009	2	-	-	One of the dizygotic twin children of Sango; father is Robin.
Maya^e^	F	Jan. 9, 1990	21	-	-	Captive-born. An original member of the Noichi group.
Gou^e^	M	1977	34	-	-	Captive-born and introduced to the Noichi group in 2011

^a^As of April 9, 2011, the beginning of this study.

^b^The number of scans in which Sango and the focal adult were within the reach of each other was divided by the total number of scans carried out in the observation days in which both Sango and the focal adult were in the outside enclosure and multiplied by 100.

^c^If the proximity value of an adult and Sango was over the average (11.91%), then the adult was classified as an affiliated adult. On the other hand, if the proximity value of an adult and Sango was under the average, then the adult was classified as an unaffiliated adult.

^d^Estimated.

^e^Because these chimpanzees were often kept in the indoor rooms and the opportunities to observe them in the outside enclosure were few, we excluded them from the participants.

**Table 6:** Information on the number of focal observation, observation days, observation time (min), and number of scans

Name	Number of observation days^a^	Number of days in which focal observations were conducted^b^	Number of focal observations	Total time of focal observations (minutes)^c^	The average number of focal observation per day^d^	The average length of focal observation per day (minutes)^e^	Number of scans	The average number of scans per day^f^
Sango	24	24	49	490	2.04	20.42	382	15.92
Robin	24	23	42	420	1.83	18.26	382	15.92
Koyuki	22	19	41	410	2.16	21.58	352	16.00
Cherry	21	21	39	390	1.86	18.57	332	15.81
Chelsea	19	19	35	350	1.84	18.42	298	15.68
Judy	21	20	38	380	1.90	19.00	348	16.57

^a^Since some chimpanzees kept in the indoor rooms on some days while Sango and her twins were in the outside enclosure, Sango's number of observation days differed from other chimpanzees.

^b^For some chimpanzees, the number of days in which focal observations were conducted differed from that of the number of observation days because there were a few days when we could not conduct focal observation but could conduct scan sampling.

^c^Since each focal sampling was conducted continuously for 10 minutes, the total time of focal observation was calculated by multiplying the number of focal observations by 10.

^d^The average number of focal observations per day was calculated by dividing the total number of focal observations by the number of days in which focal observation were conducted.

^e^The average length of focal observation per day was calculated by dividing the total time of focal observation by the number of days in which focal observations were conducted.

^f^The average number of scans per day was calculated by dividing the number of scans by the number of observation days.
